# Correction: Effects of unilateral and bilateral complex-contrast training on lower limb strength and jump performance in collegiate female volleyball players

**DOI:** 10.1371/journal.pone.0337121

**Published:** 2025-11-18

**Authors:** Beiwang Deng, Ruixiang Yan, Xinke Sui, Gesheng Lin, Shicong Ding, Duanying Li, Jian Sun

The affiliation for the fifth author is incorrect. Shicong Ding correct affiliation is: School of Athletic Training, Guangzhou Sport University, Guangzhou, Guangdong, China.

The email address for Shicong Ding’ is incorrect. The correct email address is: dingsc@gzsport.edu.cn.
