# Supplementary figures and images for: Effects of unilateral and bilateral complex-contrast training on lower limb strength and jump performance in collegiate female volleyball players
Source: PLoS One. 2025 Jun 30;20(6):e0327237. doi: 10.1371/journal.pone.0327237 (PMC12208454; doi:10.1371/journal.pone.0327237)

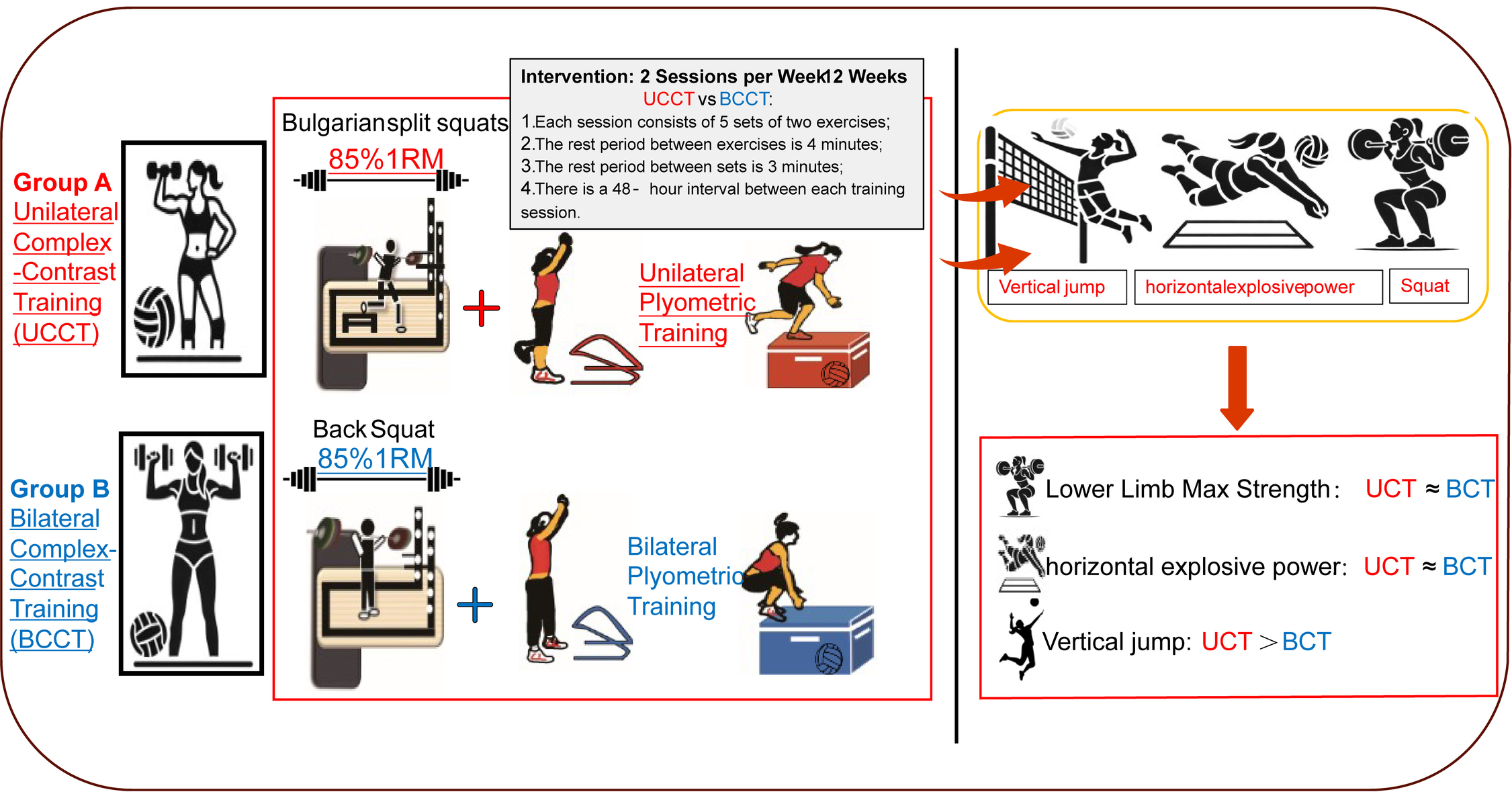

Supplement: S1 Fig — (TIF) [file pone.0327237.s002.tif]
