# Supplementary material for: Effects of unilateral and bilateral complex-contrast training on lower limb strength and jump performance in collegiate female volleyball players
Source: PLoS One. 2025 Jun 30;20(6):e0327237. doi: 10.1371/journal.pone.0327237 (PMC12208454; doi:10.1371/journal.pone.0327237)
Supplement: Attachment — Submitted filename: Plos one 15.03.2025.docx [file pone.0327237.s003.docx]

**Experimental Study on the Comparative Effects of Unilateral and Bilateral Complex Contrast Training on Lower Limb Strength and Jump Performance in Collegiate Female Volleyball Players**

As a reviewer for *PLOS ONE*, I would like to acknowledge the authors for their efforts in conducting this study on the comparative effects of unilateral and bilateral complex contrast training on lower limb strength and jump performance in collegiate female volleyball players. The topic is relevant to sports science and contributes to the understanding of training methods for athletic performance enhancement. However, while the study presents valuable insights, the statistical analysis and tables lack clarity, making it difficult to fully interpret the findings. To ensure transparency and reproducibility, I recommend a thorough revision of the results section, including clearer statistical reporting, effect sizes, confidence intervals, and better-structured tables. Additionally, enhancing the discussion with a more critical analysis of the findings in relation to existing literature would strengthen the paper. If these revisions are addressed, I believe the article has the potential to meet the journal's standards for publication.

**Title**: change the title or delete “**Experimental Study on**”

**Abstract**:

L15-18: rewrite this sentence and avoid to used upper capital in the beginning of some words

L18: “A total of 16 female volleyball players were randomly assigned to either the UCCT group (n=8) or the BCCT group (n=8)”; randomly by what ??? player position, biologic age or what ????

Add more information about participants

L20-23: rewrite this sentence or delete “to assess changes in lower limb strength, vertical jump ability, and horizontal jump ability”

Add sentence about statistical tool used in this study

The result and conclusion are not clear, please rewrite

**Keywords**: Changed keywords (avoid used keywords used in the title)

**Introduction**:

The introduction of the article *"Experimental Study on the Comparative Effects of Unilateral and Bilateral Complex Contrast Training on Lower Limb Strength and Jump Performance in Collegiate Female Volleyball Players"* lacks clarity in establishing the research gap and justification for the study. While it provides a general overview of complex contrast training (CCT) and its potential benefits, it fails to offer a strong theoretical foundation explaining why unilateral versus bilateral training may yield different outcomes. Additionally, the introduction does not sufficiently engage with existing literature to highlight inconsistencies or unresolved questions, making it difficult to understand the novelty of the study. A more structured argument, supported by a critical review of past research, would strengthen the rationale for the investigation and improve its overall impact. REWRITE

**Methods**:

L76-77: rewrite this sentence

L80: “18 participants were randomly assigned to either the UCCT group (n=9) or the BCCT group (n=9) using a random number-based randomization method” HOW ??? more information

L85: changed “Squat 1RM” by 1-RM squat and in all the text

Table 1 ?????? where is

Table 2 ?????? where is

Table 3: add abbreviation list

L188-189: Tukey post hoc, in this study you don’t need post hoc because you have 2 groups and 2 times

**Result**:

The results and tables presented in this article exhibit several shortcomings that affect their clarity and interpretability. While the tables provide numerical data on performance changes, they lack sufficient statistical indicators, such as effect sizes or confidence intervals, to contextualize the significance of the findings. Furthermore, the presentation of results does not clearly differentiate between practical and statistically significant outcomes, making it difficult to determine the real-world applicability of the findings. The authors also fail to discuss potential variability in individual responses, which is crucial in sports science research. A more detailed analysis, including graphical representations and a clearer explanation of key trends, would improve the comprehensibility and impact of the results section.

**Discussion**:

The discussion section of the article "Experimental Study on the Comparative Effects of Unilateral and Bilateral Complex Contrast Training on Lower Limb Strength and Jump Performance in Collegiate Female Volleyball Players" is weakened by the lack of clarity in the results and tables presented. Because the results are not well-structured or sufficiently detailed, the discussion fails to provide a compelling interpretation of the findings. The authors attempt to compare their results with previous studies, but without clear statistical analysis or well-presented data, these comparisons remain vague and inconclusive. Additionally, the discussion lacks a critical evaluation of potential limitations. Without a more precise and structured presentation of results, the discussion becomes speculative rather than evidence-based, making it difficult to draw meaningful conclusions or practical applications for training protocols. A more rigorous analysis and transparent data reporting are necessary to strengthen the discussion and support the study’s claims.
