# Supplementary material for: Effects of unilateral and bilateral complex-contrast training on lower limb strength and jump performance in collegiate female volleyball players
Source: PLoS One. 2025 Jun 30;20(6):e0327237. doi: 10.1371/journal.pone.0327237 (PMC12208454; doi:10.1371/journal.pone.0327237)
Supplement: Attachment — Submitted filename: Response to Reviewers.docx [file pone.0327237.s004.docx]

Dear Editors and Reviewers:

Thank you for your letter and for the reviewers’ comments concerning our manuscript entitled **“Effects of Unilateral and Bilateral Complex-Contrast Training on Lower Limb Strength and Jump Performance in Collegiate Female Volleyball Players” (ID: PONE-D-25-07993)**. On behalf of my co-authors, we thank you for allowing us to revise our manuscript. These comments are all valuable and very helpful for revising and improving our paper, and they provide important guiding significance to our research. We have studied the comments carefully and have made corrections, which we hope meet your approval.

The revised portions are marked in red in the manuscript. In the response to the reviewer, the parentheses at the end correspond to the line numbers. The main corrections in the paper and the responses to the reviewer’s comments are as follows:

**Responds to Journal Requirements:**

**Reviewer's Comment 1:**

*Please ensure that your manuscript meets PLOS ONE's style requirements, including those for file naming. The PLOS ONE style templates can be found at*

*[https://journals.plos.org/plosone/s/file?id=wjVg/PLOSOne_formatting_sample_main_body.pdf](https://journals.plos.org/plosone/s/file?id=wjVg/PLOSOne_formatting_sample_main_body.pdf) and*

*[https://journals.plos.org/plosone/s/file?id=ba62/PLOSOne_formatting_sample_title_authors_affiliations.pdf](https://track.editorialmanager.com/CL0/https://journals.plos.org/plosone/s/file?id=ba62/PLOSOne_formatting_sample_title_authors_affiliations.pdf/1/010f019660727069-6eeb2e29-9e0f-4d5d-ab43-c0f816d30718-000000/lyozq6fZvzXLdk9q0fSfZfdjo34X6w2bF1_YJiStpV4=208).*

**Response to Comment 1：**

Thank you for the reminder. We have reviewed and revised the manuscript to ensure that it adheres to the PLOS ONE formatting guidelines. The file names and structural components of the submission have also been updated in accordance with the journal’s requirements.

**Reviewer's Comment 2:**

*Thank you for stating the following financial disclosure:*

*[Guangdong Provincial Philosophy and Social Sciences Regularization Project 2022 (GD22CTY09): Research on the Coordinated Development Path of International Competitiveness in Sports in the Guangdong-Hong Kong-Macao Greater Bay Area.].*

*Please state what role the funders took in the study.  If the funders had no role, please state: ""The funders had no role in study design, data collection and analysis, decision to publish, or preparation of the manuscript.""*

*If this statement is not correct you must amend it as needed.*

*Please include this amended Role of Funder statement in your cover letter; we will change the online submission form on your behalf.*

**Response to Comment 2：**

Thank you for your careful reminder. As requested, we have added the funder role disclosure using the recommended wording:

*“The funders had no role in study design, data collection and analysis, decision to publish, or preparation of the manuscript.”*

This statement is now clearly included at the end of the revised cover letter.

**Reviewer's Comment 3:**

*Your ethics statement should only appear in the Methods section of your manuscript. If your ethics statement is written in any section besides the Methods, please delete it from any other section*

**Response to Comment 3：**

Thank you. We have revised the manuscript to ensure that the ethics statement now appears only in the Methods section. Any duplicate or misplaced instances of the ethics statement in other sections have been removed accordingly.

**Reviewer's Comment 4:**

*Please include a caption for figure 2.*

**Response to Comment 4：**

Thank you for pointing this out. We have added a caption for Figure 2 in the revised manuscript. Additionally, we have carefully reviewed all other figures to ensure that each one includes a complete and appropriately formatted caption.

**Reviewer's Comment 5:**

*Please include a copy of Table 1 and 2 which you refer to in your text on page 8.*

**Response to Comment 5：**

Thank you. We have now included the complete versions of Table 1 and Table 2 in the main manuscript as referenced on page 8. We appreciate your attention to this important formatting detail.

**Reviewer's Comment 6:**

*We notice that your tables are uploaded with the file type ' Supporting Information'. Please amend the file type to 'Table’.*

**Response to Comment 6：**

Thank you for the notice. We have amended the file type designation for Table 1 and Table 2 from ‘Supporting Information’ to ‘Table’ as requested.

**Responds to the Reviewer #1:**

**Response to General Comment:**

*As a reviewer for PLOS ONE, I would like to acknowledge the authors for their efforts in conducting this study on the comparative effects of unilateral and bilateral complex contrast training on lower limb strength and jump performance in collegiate female volleyball players. The topic is relevant to sports science and contributes to the understanding of training methods for athletic performance enhancement. However, while the study presents valuable insights, the statistical analysis and tables lack clarity, making it difficult to fully interpret the findings. To ensure transparency and reproducibility, I recommend a thorough revision of the results section, including clearer statistical reporting, effect sizes, confidence intervals, and better-structured tables. Additionally, enhancing the discussion with a more critical analysis of the findings in relation to existing literature would strengthen the paper. If these revisions are addressed, I believe the article has the potential to meet the journal's standards for publication.*

**Response to General Comment：**

We sincerely thank the reviewer for their encouraging and thoughtful comments. In response, we have substantially revised the Results section to improve clarity, statistical transparency, and interpretability. Specifically, we have:

(1) Reorganized and reformatted all tables for clarity and completeness;
(2) Added effect sizes (η²p) and 95% confidence intervals where appropriate;
(3) Enhanced the accompanying text to clearly describe the direction and significance of changes;
(4) Added visual figures to better communicate individual and group-level trends.

In addition, the Discussion section has been revised to include a more balanced and critical comparison with existing literature, and to ensure that interpretations are consistent with our results. We appreciate your recognition of the study’s potential and your helpful guidance in improving the manuscript.

**Reviewer's Comment 1:**

*change the title or delete “Experimental Study on.*

**Response to Comment 1：**

We thank the reviewer for this valuable suggestion. In accordance with your recommendation, we have removed “Experimental Study” from the title. The revised title is now:**"Effects of Unilateral and Bilateral Complex-Contrast Training on Lower Limb Strength and Jump Performance in Collegiate Female Volleyball Players.**

**Reviewer's Comment 2:**

*Lines 15–18: Please rewrite this sentence and avoid using capital letters at the beginning of some words.*

**Response to Comment 2：**

Thank you for the suggestion. We have revised the sentence to improve clarity and removed the inappropriate capitalization. (Lines 14–19)

**Reviewer's Comment 3:**

*A total of 16 female volleyball players were randomly assigned to either the UCCT group (n=8) or the BCCT group (n=8)”; randomly by what ??? player position, biologic age or what ????.*

**Response to Comment 3：**

Thank you for your important question. We have clarified the randomization method in the revised manuscript. Specifically, we added that participants were randomly assigned using a random number-based randomization method in SPSS software. (Lines 19–22)

**Reviewer's Comment 4:**

*Add more information about participants.*

**Response to Comment 4：**

Thank you for the suggestion. We have added detailed information regarding the participants’ age, height, body mass, and training experience, along with a CONSORT flow diagram to illustrate the recruitment and allocation process. (Lines 166–167)

**Reviewer's Comment 5:**

*rewrite this sentence or delete “to assess changes in lower limb strength, vertical jump ability, and horizontal jump ability.*

**Response to Comment 5：**

Thank you for the suggestion. We have deleted the sentence as advised to improve clarity and avoid redundancy.

**Reviewer's Comment 6:**

*Add sentence about statistical tool used in this study.*

**Response to Comment 6：**

Thank you for the suggestion. We have added a sentence in the Abstract to indicate the statistical tool and analysis method used in this study:

“Statistical analysis was conducted using JASP version 0.18.3.0 with a 2 (group) × 2 (time) two-way repeated measures ANOVA.”(Lines 24–25)

**Reviewer's Comment 7:**

*The result and conclusion are not clear, please rewrite.*

**Response to Comment 7：**

Thank you for your constructive feedback. In response, we have revised the Results and Conclusion in the Abstract to improve clarity and conciseness. (Lines 25–31)

**Reviewer's Comment 8:**

*Keywords: Changed keywords (avoid used keywords used in the title).*

**Response to Comment 8：**

Thank you for the suggestion. We have revised the keywords to avoid redundancy with the title. The new keywords are: Unilateral Training; Bilateral Training; Resistance Training; Female Athletes. (Line 32)

**Reviewer's Comment 9:**

*The introduction of the article "Experimental Study on the Comparative Effects of Unilateral and Bilateral Complex Contrast Training on Lower Limb Strength and Jump Performance in Collegiate Female Volleyball Players" lacks clarity in establishing the research gap and justification for the study. While it provides a general overview of complex contrast training (CCT) and its potential benefits, it fails to offer a strong theoretical foundation explaining why unilateral versus bilateral training may yield different outcomes. Additionally, the introduction does not sufficiently engage with existing literature to highlight inconsistencies or unresolved questions, making it difficult to understand the novelty of the study. A more structured argument, supported by a critical review of past research, would strengthen the rationale for the investigation and improve its overall impact. REWRITE*

**Response to Comment 9：**

We sincerely thank the reviewer for this constructive and detailed comment. In response, we have thoroughly rewritten the Introduction section (Lines 34–111) to enhance its clarity, structure, and theoretical rigor. Specifically:

(1) We clearly defined the research gap by pointing out the lack of studies directly comparing unilateral and bilateral complex-contrast training (UCCT vs. BCCT) in collegiate female volleyball players.
(2) We expanded the theoretical foundation by explaining the distinct neuromuscular and biomechanical adaptations associated with unilateral and bilateral training modalities, including references to the cross-education effect, interlimb coordination, load stimulus distribution, and movement specificity.
(3) We provided a critical review of the literature, summarizing both supporting and conflicting findings regarding the effectiveness of unilateral and bilateral resistance training in improving strength and jump performance.
(4) We highlighted the novelty and practical significance of this study in addressing unresolved questions and guiding targeted strength and conditioning strategies for female volleyball athletes.

We believe these revisions have significantly improved the clarity and scientific justification of the study, and we appreciate your valuable feedback.

**Reviewer's Comment 10:**

*L76-77: rewrite this sentence.*

**Response to Comment 10：**

Thank you for your suggestion. We have revised the sentence to improve clarity and readability. The updated sentence now reads:

*“The study adopts a randomized parallel control trial design, aiming to analyze the effects of UCCT and BCCT on lower limb strength and jump performance in collegiate female volleyball players, providing scientific evidence for training and optimizing jump ability.” (Lines 114–116)*

**Reviewer's Comment 11:**

*18 participants were randomly assigned to either the UCCT group (n=9) or the BCCT group (n=9) using a random number-based randomization method” HOW ??? more information.*

**Response to Comment 11：**

Thank you for pointing this out. To clarify the randomization procedure, we have revised the sentence to include more detail. The updated text now reads:

“After familiarizing themselves with the test procedures and completing pre-testing, 18 participants were randomly assigned to either the UCCT group (n=9) or the BCCT group (n=9) using a random number-based randomization method in SPSS software.” (Lines 127–129)

This provides a clearer description of the randomization process used in the study.

**Reviewer's Comment 12:**

*changed “Squat 1RM” by 1-RM squat and in all the text.*

**Response to Comment 12：**

Thank you for your suggestion. We have carefully revised the manuscript and consistently replaced all instances of “Squat 1RM” with “1-RM squat” to ensure terminology consistency across the entire text.

**Reviewer's Comment 13:**

*Table 1 ?????? where is.*

**Response to Comment 13：**

We sincerely apologize for the oversight. Table 1 was mistakenly omitted in the previous submission. We have now added Table 1 to the revised manuscript, which presents the baseline characteristics of the participants. (Lines 166–167)

**Reviewer's Comment 14:**

*PTable 2 ?????? where is.*

**Response to Comment 14：**

We apologize for the omission. Table 2 was inadvertently left out in the previous submission. It has now been added to the revised manuscript and presents the formal training experimental plan for both the UCCT and BCCT groups. (Lines 192–193)

**Reviewer's Comment 15:**

*add abbreviation list.*

**Response to Comment 15：**

Thank you for the suggestion. We have added a list of abbreviations at the end of the manuscript to improve clarity and facilitate reader understanding. (Lines 438–439)

**Reviewer's Comment 16:**

*L188-189: Tukey post hoc, in this study you don’t need post hoc because you have 2 groups and 2 times.*

**Response to Comment 16：**

Thank you for this statistical clarification. We have removed the Tukey post hoc analysis from the manuscript as it is not necessary for a 2 (group) × 2 (time) design with only two levels per factor. (Deletion from Lines 188–189)

We sincerely appreciate the reviewer’s careful attention to statistical detail, which has helped improve the precision of our analysis.

**Reviewer's Comment 17:**

*The results and tables presented in this article exhibit several shortcomings that affect their clarity and interpretability. While the tables provide numerical data on performance changes, they lack sufficient statistical indicators, such as effect sizes or confidence intervals, to contextualize the significance of the findings. Furthermore, the presentation of results does not clearly differentiate between practical and statistically significant outcomes, making it difficult to determine the real-world applicability of the findings. The authors also fail to discuss potential variability in individual responses, which is crucial in sports science research. A more detailed analysis, including graphical representations and a clearer explanation of key trends, would improve the comprehensibility and impact of the results section.*

**Response to Comment 17：**

We sincerely thank the reviewer for this thorough and insightful evaluation. In response, we have made the following comprehensive revisions to enhance the clarity, interpretability, and practical relevance of our results section (Lines 273–311):

(1) We have corrected formatting issues in the results and tables, which were previously caused by submission-related layout inconsistencies. The tables have been carefully reviewed and revised to ensure alignment between data and descriptions.
(2) Effect sizes have been added to the relevant tables to provide a better understanding of the magnitude of differences.
(3) Where appropriate, we have included confidence intervals to support the interpretation of statistical findings and provide greater context.
(4) We have included graphical representations (figures) to illustrate individual-level pre–post changes, helping to convey variability in participant responses and enhance the real-world applicability of the results.
(5) Additionally, we have revised the narrative description of the results to more clearly distinguish between statistical significance and practical relevance, in line with best practices in sports science research.
(6) These revisions appear in the Results section and corresponding tables and figures. We appreciate your guidance in improving the quality and scientific transparency of our findings.

Figure 4：Individual Changes in 1-RM Squat Following Training Intervention

Note: Mean ± 95% CI; *P<0.05; **P<0.01.

**Reviewer's Comment 18:**

*The discussion section of the article "Experimental Study on the Comparative Effects of Unilateral and Bilateral Complex Contrast Training on Lower Limb Strength and Jump Performance in Collegiate Female Volleyball Players" is weakened by the lack of clarity in the results and tables presented. Because the results are not well-structured or sufficiently detailed, the discussion fails to provide a compelling interpretation of the findings. The authors attempt to compare their results with previous studies, but without clear statistical analysis or well-presented data, these comparisons remain vague and inconclusive. Additionally, the discussion lacks a critical evaluation of potential limitations. Without a more precise and structured presentation of results, the discussion becomes speculative rather than evidence-based, making it difficult to draw meaningful conclusions or practical applications for training protocols. A more rigorous analysis and transparent data reporting are necessary to strengthen the discussion and support the study’s claims.*

**Response to Comment 18：**

We sincerely appreciate the reviewer’s detailed and constructive feedback. In response, we have substantially rewritten the Discussion section (Lines 316–384) to improve its clarity, logical flow, and scientific rigor. The revised version now includes:

(1) A clear interpretation of each outcome variable, linking observed improvements to statistical results, including group-specific changes and effect sizes.
(2) A comparative analysis with prior literature, with attention to both concordant and contradictory findings. We provide physiological and neuromechanical explanations—such as the specificity principle, neuromuscular coordination, and cross-education effect—to interpret observed differences between UCCT and BCCT.
(3) A detailed evaluation of practical implications, emphasizing the transferability of unilateral training to bilateral performance and its relevance to volleyball-specific motor demands.
(4) An implicit acknowledgment of individual variability and suggestions for program design optimization based on the training goal (e.g., short-term explosive peak vs. long-term strength development).
(5) A more critical view of potential limitations, such as the relatively small sample size and possible short-term effect duration, is also integrated into the interpretation.

We believe that these revisions have considerably strengthened the depth, clarity, and applied value of the Discussion section. Thank you again for pointing out this important area for improvement.

**Responds to the Reviewer #2:**

**Response to General Comment:**

*Thank you for the opportunity to review this manuscript. In general, I believe the introduction needs a deeper discussion of more relevant topics to set the scene for the study, and the discussion needs to be much more balanced. I have made specific comments below.*

**Response to General Comment：**

Thank you for your insightful feedback. We have thoroughly revised the Introduction to expand upon relevant theoretical and empirical literature, particularly on the rationale behind combining strength and plyometric training and the role of CCT. The revised version also provides a clearer justification for comparing unilateral and bilateral execution modes. The Discussion has likewise been updated to present a more balanced interpretation of our findings in light of prior studies. We appreciate your suggestions, which significantly improved the coherence and depth of our argumentation.

**Reviewer's Comment 1:**

*Line 22 – suggest writing 1RM in full.*

**Response to Comment 1：**

Thank you for the suggestion. We have revised the text to use the full form “1-RM squat” for improved clarity and consistency. In addition, we have carefully reviewed the entire manuscript to ensure that this terminology is used consistently throughout. (Line 24)

**Reviewer's Comment 2:**

*Line 25 – would be good to state the direction of the group X time effect here to highlight ¬which intervention was more effective.*

**Response to Comment 2：**

Thank you for the helpful suggestion. We have revised the sentence to clearly indicate the direction of the group × time interaction effect. (Lines 25–30)

**Reviewer's Comment 3:**

*Line 43 – suggest also adding some commentary around power/plyometric training here as a another potential method of improving performance. This will lead into the next paragraph where you then talk about how they can be combined using CCT.*

**Response to Comment 3：**

Thank you for the suggestion. We have added commentary on power and plyometric training as complementary strategies for improving jump performance, which helps build a logical lead-in to the following discussion of complex-contrast training. (Lines 44–58)

**Reviewer's Comment 4:**

*Line 44 to 48 – this section is quite brief. It would be good to describe some applied research examining similar outcomes using CCT. Schneiker et al., 2023 and Luders et al., 2024 are two recent studies that investigated the topic that could be discussed.*

**Response to Comment 4：**

Thank you for your valuable suggestion. We agree that recent applied studies provide important empirical support for the effectiveness of CCT. In response, we have expanded the relevant section and have incorporated both Schneiker et al. (2023) and Luders et al. (2024) to highlight applied findings on strength and explosive performance improvements using complex training approaches. (Lines 51–79)

**Reviewer's Comment 5:**

*Line 46 – can you provide an example of what this might look like? (i.e., back squat into box jumps, or something like that)*

**Response to Comment 5：**

Thank you for the helpful suggestion. In response, we have expanded the relevant paragraph to include specific examples illustrating each type of complex training, such as back squats followed by standing long jumps or box jumps followed by high-load squats. These examples help clarify the practical structure of each training modality. (Lines 63–73)

**Reviewer's Comment 6:**

*Line 47 – need to expand on PAPE in more detail as it is what underpins the potential benefit of CCT.*

**Response to Comment 6：**

Thank you for your helpful suggestion. We have expanded the explanation of the PAPE (post-activation potentiation effect) mechanism to clarify how it contributes to the benefits of CCT. Specifically, we now state that PAPE enhances the force and contraction speed of subsequent low-load exercises, thereby improving the effectiveness of explosive training. (Lines 73–75)

**Reviewer's Comment 7:**

*Line 49 – this sentence doesn’t really suggest that CCT is worth investigating (i.e., why not just do plyometric exercises then strength exercises?). Suggest using the papers mentioned above to build a stronger rationale for CCT in your population group.*

**Response to Comment 7：**

Thank you for this valuable comment. We have revised the paragraph (Lines 75–82) to more clearly justify the investigation of CCT in our study. In addition to highlighting recent meta-analytical findings showing that CCT leads to greater improvements in maximal strength and vertical jump performance compared to other complex training models, we now emphasize the underlying role of the post-activation performance enhancement (PAPE) mechanism. CCT takes advantage of PAPE by alternating high-load and low-load exercises within a single session, thereby enhancing the neuromuscular response and improving the effectiveness of subsequent explosive movements. Together, these physiological and empirical arguments strengthen the rationale for examining CCT in volleyball athletes. (Lines 73–79)

**Reviewer's Comment 8:**

*Line 56 to 63 – this section uses very broad language, and the point you make isn’t particularly clear. It would be better to refer to some literature looking at applied outcomes in response to unilateral and bilateral strength training as a way to highlight what the benefits of both are. The meta-analysis by Zhang et al., 2023 on the topic would be a good starting point.*

**Response to Comment 8：**

Thank you for the suggestion. We have revised the section to clarify the comparative advantages of unilateral and bilateral training, and to improve the specificity of our argument. As recommended, we also incorporated Zhang et al. (2023) to strengthen the applied rationale with recent meta-analytic evidence.(Lines 87–99)

**Reviewer's Comment 9:**

*Line 64 to 71 – would be good to mention in here that (at least to my knowledge) no study has compared unilateral to bilateral CCT, which makes this a rather novel project.*

**Response to Comment 9：**

Thank you for this insightful suggestion. We have revised the paragraph to explicitly state that, to our knowledge, no prior studies have systematically compared unilateral and bilateral CCT. We especially appreciate this comment, as it encouraged us to clearly highlight the novelty of our research questio. (Lines 100–102)

**Reviewer's Comment 10:**

*Line 71 – this hypothesis is different to what you started in your pre-registration, whereby you said you were exploring the two modalities to simply see what was better. This hypothesis/aim should be the same as your pre-registration.*

**Response to Comment 10：**

Thank you for pointing this out. To ensure consistency with our pre-registered study protocol, we have revised the statement of our hypothesis. It now reflects the original exploratory aim—to examine the comparative effects of UCCT and BCCT—without implying a directional expectation that was not specified in the pre-registration. (Lines 109–111)

**Reviewer's Comment 11:**

*Line 79 – can you also describe where in the competitive season this was? Was it pre-season, in season, etc. Moreover, can you speak to their training in the prior months before the study. Were they regularly conducting resistance training?*

**Response to Comment 11：**

Thank you for this valuable suggestion. We have revised the Experimental Approach to the Problem section to clarify that the study was conducted during the early season phase. We also added a detailed description of the participants’ training routines in the months prior to the intervention. This included regular volleyball-specific practices and conditioning sessions involving both bilateral and unilateral lower limb exercises. All participants were familiar with resistance training techniques prior to the study, and their typical weekly routine was maintained throughout the intervention to ensure ecological validity. A weekly training overview has been added as Table 2 to support this description. (Lines 114–125)

Table 1 Weekly training program

|  | Mon | Tue | Wed | Thu | Fri | Sat | Sun |
| --- | --- | --- | --- | --- | --- | --- | --- |
| AM | / | Technical | / | Tactical | Training situations (1-on-1) | Match / Scrimmage | Rest |
| PM | CCT Intervention (UCCT/BCCT) | / | CCT Intervention (UCCT/BCCT) | / | / | Rest or Active Recovery | / |

Note: am: 9:00–11:30; pm: 15:00–17:30.

**Reviewer's Comment 12:**

*Line 81 – can you elaborate on the randomisation method? Was this an online tool?*

**Response to Comment 12：**

Thank you for the comment. We have clarified the randomisation procedure in the revised manuscript. Specifically, participants were randomly assigned to either group using a random number-based randomization method implemented in SPSS software. (Lines 126–128)

**Reviewer's Comment 13:**

*Line 91 – need to provide more information on why you thought an effect size of two very similar interventions would be medium? This seems unrealistic to expect.*

**Response to Comment 13：**

Thank you for raising this important question. We have revised the relevant section to provide additional details on our sample size calculation and the rationale for selecting a medium effect size. Specifically, the estimated effect size (f = 0.5) was based on findings from a previous study involving an 8-week explosive training intervention in young male football players, which reported medium effects on neuromuscular performance. Moreover, we have fully described the G*Power parameters used for our estimation, including test type, power, number of groups and measurements, and epsilon correction, to ensure methodological transparency. (Lines 138–144)

**Reviewer's Comment 14:**

*Line 93 – was this a convenience sample? If so, this needs to be stated.*

**Response to Comment 14：**

Thank you for raising this point. We confirm that the sample was not recruited using a convenience sampling method. Instead, participants were randomly selected from a full pool of eligible female collegiate volleyball athletes who met the predefined inclusion criteria. This clarification has been added to the revised manuscript, along with a CONSORT flow diagram to illustrate the recruitment and allocation process. (Lines 144–145)

Figure 1 CONSORT flow diagra

**Response to Comment 15：**

*Line 93 – can you provide more information on the level of the athletes? What level of competition were they competing in?*

**Response to Comment 15：**

Thank you for the suggestion. We have added a description of the athletes’ competitive level to the revised manuscript. All participants were members of the same university volleyball team and regularly competed in regional-level events, including the B subgroup of the Elite Division in the Guangdong Collegiate Volleyball League. In addition, they were selected to compete in the 12th Guangdong University Games, a provincial competition held every four years, indicating a relatively high level of performance among collegiate athletes in China. (Lines 150–154)

**Reviewer's Comment 16:**

*Line 103 – can you speak to the experience of the athletes with these exercises specifically? Had they all been doing squats and Bulgarian split squats in their training, or were they all new exercises?*

**Response to Comment 16：**

Thank you for the comment. We have clarified this point in the revised manuscript. Prior to the intervention, the athletes' regular weekly training included conditioning sessions that incorporated exercises such as bilateral squats and Bulgarian split squats, depending on the phase of their training cycle. Therefore, all participants were familiar with both bilateral and unilateral lower limb exercises before the study began. (Lines 120–124)

**Reviewer's Comment 17:**

*Line 104 – can you add the range of attendance % for both groups in brackets here as well.*

**Response to Comment 17：**

Thank you for the suggestion. We have added the range of training adherence percentages for both groups in the revised manuscript. The UCCT group had an adherence rate of 94% (range: 87.5%–100%), and the BCCT group had an adherence rate of 93% (range: 87.5%–100%). (Lines 159–160)

**Reviewer's Comment 18:**

*Line 120 – who provided/supervised the intervention? Or was it conducted alone?*

**Response to Comment 18：**

Thank you for the comment. We have clarified this point in the revised manuscript. All training sessions were supervised by two Certified Strength and Conditioning Specialists (CSCS), who ensured proper exercise technique, protocol adherence, and training safety throughout the intervention. (Lines 176–178)

**Reviewer's Comment 19:**

*Line 125 – can you also provide the rest between the conditioning activity and the plyometric activity? And in this section it would be good to outline why you chose different plyometric exercises for each group.*

**Response to Comment 19：**

Thank you for this thoughtful comment. We have revised the manuscript to include the rest intervals used in the training protocol—specifically, 3 minutes between sets and 4 minutes between the resistance and plyometric components (Lines 183–184). We also clarified the rationale for selecting different plyometric exercises for each group: exercises were chosen to incorporate both vertical and horizontal movement patterns, aiming to enhance volleyball-specific jump performance by addressing multidirectional demands of the sport (Lines 180–190).

**Reviewer's Comment 20:**

Line 129 – can you also list the days for training and games, as you did above with the gym sessions.

**Response to Comment 20：**

Thank you for the suggestion. We have included a detailed weekly training schedule in the revised manuscript, which lists the specific days for strength training, volleyball-specific practices, and matches. This information is presented in the “Weekly Training Program” table for clarity and ease of reference. (Lines 118–125)

Table 1 Weekly training program

|  | Mon | Tue | Wed | Thu | Fri | Sat | Sun |
| --- | --- | --- | --- | --- | --- | --- | --- |
| AM | / | Technical | / | Tactical | Training situations (1-on-1) | Match / Scrimmage | Rest |
| PM | CCT Intervention (UCCT/BCCT) | / | CCT Intervention (UCCT/BCCT) | / | / | Rest or Active Recovery | / |

Note: am: 9:00–11:30; pm: 15:00–17:30.

**Reviewer's Comment 21:**

*Line 138 to 146 – can you provide a bit more information here on the instructions provided to participants, and whether verbal encouragement was used.*

**Response to Comment 21：**

Thank you for the suggestion. We have revised the manuscript to clarify that consistent verbal encouragement (“Jump as high as you can!”) was provided during each attempt to promote maximal performance. This instruction was applied uniformly across all participants to ensure standardized motivational input. (Lines 214–216)

**Reviewer's Comment 22:**

*Line 150 – would be good to provide some additional context as to what the purpose of this measure is.*

**Response to Comment 22：**

Thank you for this helpful comment. We have expanded our description of the Eccentric Utilization Rate (EUR) to clarify its purpose. Specifically, we now explain that EUR reflects an athlete’s ability to utilize the stretch-shortening cycle (SSC) in explosive movements. A higher EUR indicates more effective use of elastic energy and neuromuscular potentiation during the eccentric phase to enhance concentric force output—an ability that is particularly relevant to improving jump performance and lower-limb explosiveness. (Lines 220–224)

**Reviewer's Comment 23:**

*Line 152 – as above, could you provide the verbal instructions that were given to participants? And were there toes or heels placed on the starting line?*

**Response to Comment 23：**

Thank you for the helpful comment. We have expanded the SLJ test description to include standardized verbal instructions and clarify foot placement. Participants were instructed to stand with their toes just behind the starting line, without touching or crossing it. Before each attempt, the following standardized instruction was given: “Jump as far forward as you can using both legs, land with control, and avoid crossing the start line before take-off.” This information has been added to the revised manuscript (Lines 225–232).

**Reviewer's Comment 24:**

*Line 159 – in the training program you imply that the UCCT group uses 85% of their Bulgarian split squat 1RM. Was this measured? And if so, how?*

**Response to Comment 24：**

Thank you for pointing out this oversight. We have revised the manuscript to clarify that the 1-RM for the Bulgarian split squat (BSS) was directly measured prior to the intervention using the same testing protocol as the back squat, in accordance with NSCA guidelines. Detailed procedures have been added to the Methods section, including load progression, movement standards (e.g., 90-degree knee angle), and safety measures. This ensures that the training intensities prescribed for both groups were based on accurately assessed 1-RM values. (Lines 241–247)

**Reviewer's Comment 25:**

*Line 170 – what specific measures/data were used to calculate these?*

**Response to Comment 25：**

Thank you for your question. We have clarified this in the revised manuscript (Lines 257–260). ICCs were calculated using a two-way mixed-effects model [ICC(3,1)] based on absolute agreement across two baseline trials, using JASP software. Coefficients of variation (CVs) were computed as the ratio of the standard deviation to the mean from the same two baseline trials and expressed as percentages. These additions ensure clarity on the origin and calculation method of the reliability indices.

**Reviewer's Comment 26:**

*General comment – throughout the results it would be good to highlight the direction of changes. For example, both groups improved XX, UCCT saw larger improvements in XX, etc.*

**Response to Comment 26：**

Thank you for this helpful general suggestion. We have revised the Results section to more clearly highlight the direction and magnitude of change for each key outcome. For each performance indicator, we now state whether both groups improved, and when applicable, emphasize that the UCCT group demonstrated greater improvements, supported by statistical significance and effect sizes. This adjustment enhances the clarity and interpretability of the results. (Lines 272–310)

**Reviewer's Comment 27:**

*Table 4 – The post-test column for the BCT group looks like it has incorrect data? It would be good to present the mean change and 95% CI for the mean change for both groups here somewhere. Could also do the between group mean difference and 95% CI's.*

**Response to Comment 27：**

Thank you for pointing this out. We identified and corrected a formatting error in Table 4 that caused data for the BCCT group post-test values to be misaligned. This has now been rectified and the table has been carefully verified. In addition, we have updated the results presentation to include the mean change and 95% confidence intervals for both groups. We also redesigned the visualizations for each outcome variable to illustrate pre–post changes with confidence intervals, thereby improving the clarity and interpretability of the findings. (Lines 311–314)

Figure 4：Individual Changes in 1-RM Squat Following Training Intervention

Note: Mean ± 95% CI; *P<0.05; **P<0.01.

**Reviewer's Comment 28-34:**

*General comment: I believe the discussion should be changed significantly. It currently reads like an advertisement for single leg training, rather than providing an accurate and balanced discussion of the literature. I would suggest tempering your language significantly throughout, and just focusing on the results of your study rather than looking to adjacent areas of research to highlight the benefits of unilateral CCT. I have also made some more minor comments below, that may not be relevant once you make larger changes.*

*Line 227 – this first sentence is out of place here.*

*Line 228 to 234 – similar to my general comment in the results, you need to be more descriptive. And there is no need to repeat effect sizes etc. here.*

*Line 235 – dio both of these studies use CCT? It looks like one was just RT?*

*Line 241 – the review you refer to doesn’t show this? Could look for more relevant citations or be more speculative in your commentary (as this is a speculative statement)*

*Line 243 – this study looked at running, not unilateral resistance training. Remove this sentence.*

*Line 246 – this citation shows bilateral training results in more quadriceps activity than unilateral training, so this doesn’t support this statement. Suggest editing line 246 to 249 to more accurately reflect the research and be more cautious in your interpretation of your results.*

**Response to Comment 28-34：**

Thank you for your detailed and constructive feedback. In response, we have substantially revised the Discussion section to ensure a more balanced, objective, and evidence-based interpretation of the results. Specifically:

(1) We have moderated the tone throughout and removed speculative or overstated language related to unilateral training.
(2) The revised discussion focuses primarily on interpreting the findings of the current study, rather than drawing from adjacent or unrelated literature.
(3) References that were not directly relevant or did not support the associated claims have been removed or replaced.
(4) Paragraphs discussing non-significant results or controversial physiological mechanisms (e.g., acute hormonal responses) have been deleted to maintain scientific accuracy and relevance.
(5) Methodological clarifications, such as the testing protocol for Bulgarian split squat (BSS) 1-RM, have been included in the Methods section. However, as no post-test BSS data were collected, this measure was not analyzed as an outcome and was therefore not discussed.

These changes aim to present a more cautious and focused discussion aligned with the study’s actual findings and limitations. (Lines 316–383)

**Reviewer's Comment 35:**

*Also need to comment on the low sample size, and the fact that these findings may not generalise beyond this population. And that you did not compare this intervention to a “traditional” training program. As such, it is unclear whether these results are due to CCT or due to the unilateral exercises. Also, if you did not assess Bulgarian split squat 1Rm that should be noted as a limitation, as how do you know they were at 85% of their 1RM?*

**Response to Comment 35：**

Thank you for your comprehensive and constructive suggestions. In response, we have revised the Limitations section to acknowledge the relatively small sample size and the lack of a comparison group using traditional resistance training, both of which limit the strength of causal inferences. (Lines 398–405)

Regarding the Bulgarian split squat 1-RM, we confirm that it was assessed using a standardized testing protocol, as now detailed in the Methods section. Since this information is clearly presented there, we did not restate it in the limitations.

**Responds to the Reviewer #3:**

**Response to General Comment:**

*This study aimed to compare the effects of unilateral and bilateral complex training on lower limb strength and jump ability in female college volleyball athletes. While the paper is well-discussed, there are several points that require clarification or editing for improved clarity.*

**Response to General Comment：**

We sincerely thank the reviewer for the positive overall evaluation of our manuscript. In response to the suggestions for improved clarity, we have carefully revised several parts of the manuscript. For example, we corrected terminology in Table 1 (changing “Weight” to “Body mass”) and clarified the team background and training consistency of all participants. Additional adjustments were made to ensure the accuracy of data presentation and overall readability. We truly appreciate your comments, which helped us further improve the clarity and precision of the final version.

**Reviewer's Comment 1:**

*L81: Are 9 participants per group enough for your study’s randomized parallel-controlled trial design? A larger sample size would strengthen the validity of the study results and provide more robust conclusions.*

**Response to Comment 1：**

Thank you for raising this important point. We have addressed this concern in the revised Limitations section (Lines 398–401), noting that the relatively small sample size (n = 16) may have reduced statistical power, increased the likelihood of Type II error, and limited the generalizability of the findings. We also recommend that future studies recruit larger samples to enhance the robustness and external validity of the results.

**Reviewer's Comment 2:**

*L91: Given that the power was set at 0.5 in the G-power analysis, it is essential to consider if this value was based on previous research in the field. Can the authors provide a relevant citation that justifies the choice of this power size for determining the sample size in the study? Clarifying the rationale behind selecting this specific power level would strengthen the validity of the sample size calculation.*

**Response to Comment 2：**

Thank you for this thoughtful and detailed suggestion. We have now added a relevant citation to support our choice of effect size (f = 0.5), which was based on a previous study using a similar training intervention and sample size estimation approach. In addition, we have expanded the description of the G*Power parameters used in our calculation, including test type, power, sphericity correction, and number of groups and measurements. We greatly appreciate your suggestion, which helped us improve the clarity and methodological transparency of this section. (Lines 138–144)

**Reviewer's Comment 3:**

*The study's limitation of a small sample size should be acknowledged, especially considering that only 8 athletes in each group completed both the training and assessments. This information highlights the importance of interpreting the results with caution due to the relatively limited number of participants who fully participated in the study.*

**Response to Comment 3：**

Thank you for your valuable comment. We have addressed the issue of small sample size in two places within the revised manuscript. First, in the Discussion section (Lines 320–322), we acknowledged that the lack of statistical significance in certain intergroup comparisons (e.g., SJ and DJ) may be attributable to the limited number of participants (8 per group), which could have reduced statistical power. Second, in the Limitations section (Lines 398–401), we explicitly state that the small final sample size may have increased the risk of Type II error and limited the generalizability of the findings. We appreciate your feedback, which helped us clarify this methodological limitation more transparently.

**Reviewer's Comment 4:**

*Table 1.: Please change “Weight” with “Body mass.*
**Response to Comment 4：**

Thank you for your careful observation. Yes, the intended meaning was the number of training sets performed for each exercise. To improve clarity and avoid confusion, we have revised the label “Number of group” to “Number of sets” in Table 2. We appreciate your attention to detail, which helped us improve the precision of the training protocol presentation. (Lines 191–192)

**Reviewer's Comment 5:**

*Without equating the total training volume between the experimental groups, it becomes challenging to attribute any observed differences in outcomes solely to the type of training.*

*Both resistance and plyometric training exercises should be equated in volume.*

*Resistance: number of sets × total number of repetitions × load*

*Plyometric: number of foot contacts*

**Response to Comment 5：**

Thank you for this important and insightful comment. We fully agree that the lack of precise volume matching between the UCCT and BCCT protocols represents a key methodological limitation. As suggested, we have added a detailed statement to the Limitations section (Lines 390–397), noting that although both groups trained at the same relative intensity (85% 1-RM) with matched sets and repetitions, the biomechanical nature of unilateral versus bilateral exercises likely resulted in unavoidable discrepancies in actual workload.

We also appreciate the opportunity to reflect on how this challenge has been approached in previous studies. Several works (e.g., DOI: 10.1519/JSC.0000000000001096; DOI: 10.1519/JSC.0000000000003659; DOI: 10.1519/JSC.0000000000001962) have adopted similar matching strategies without fully equating unilateral and bilateral workloads, and we developed our protocol with reference to these established practices. Nevertheless, we now explicitly acknowledge this issue as a limitation of the present study, and we thank you again for highlighting this important aspect.

**Reviewer's Comment 6:**

*According to Table 2, the training intervention lacks progressive overload, as indicated by the absence of intensity, volume, or complexity adjustments over the 8-week training period. Without systematically increasing the training stimulus to challenge the athletes' physiological capacities, the potential for optimal improvements in explosive power may be limited.*

**Response to Comment 6：**

Thank you for this valuable comment. We fully agree that the absence of progressive overload is an important limitation that may have affected the training outcomes. In the revised Limitations section, we now explicitly note that the 8-week intervention did not include progressive increases in load, volume, or movement complexity. We appreciate your suggestion, which helped us more accurately describe the constraints of our training protocol and highlight important considerations for future program design. (Lines 396–397)

**Reviewer's Comment 7:**

*128-129: Were all the athletes from the same volleyball team? Did they participate in the same volleyball training sessions?*

**Response to Comment 7：**

Thank you for your question. We have clarified in the revised manuscript that all participants were members of the same university volleyball team and competed together in regional-level events, including the B subgroup of the Elite Division in the Guangdong Collegiate Volleyball League. They were also selected for the 12th Guangdong University Games, a provincial competition held every four years (Lines 150–154). In addition, we have included a “Weekly Training Program” table outlining their typical volleyball-specific training schedule, confirming that all athletes participated in the same team sessions. (Lines 135–136)

Table 1 Weekly training program

|  | Mon | Tue | Wed | Thu | Fri | Sat | Sun |
| --- | --- | --- | --- | --- | --- | --- | --- |
| AM | / | Technical | / | Tactical | Training situations (1-on-1) | Match / Scrimmage | Rest |
| PM | CCT Intervention (UCCT/BCCT) | / | CCT Intervention (UCCT/BCCT) | / | / | Rest or Active Recovery | / |

Note: am: 9:00–11:30; pm: 15:00–17:30.

**Reviewer's Comment 8:**

*L139-146: Please describe also how the participants landed.*

**Response to Comment 8：**

Thank you for this helpful comment. We have revised the manuscript to specify that participants were required to land with both feet simultaneously while flexing their knees to absorb the impact. Trials were repeated if participants landed asymmetrically, missed the mat, or failed to maintain correct posture. These additions, included in the revised text, help clarify the landing protocol and reinforce the consistency of movement execution during testing. (Lines 209–218)

**Reviewer's Comment 9:**

*Table 4: Mean values for BCCT are missing; there is only SD.*

**Response to Comment 9：**

Thank you for your careful observation. We sincerely apologize for this oversight, which was due to a formatting error that caused the mean values for the BCCT group to shift out of place in Table 4. This has now been corrected in the revised manuscript (Lines 311–314), and we have carefully reviewed the table to ensure the accuracy and clarity of all reported values. We appreciate your attention to detail, which helped us improve the quality of data presentation.

**Reviewer's Comment 10:**

*Table 4: Please change all instances of "BCT" to "BCCT" and "UCT" to "UCCT" for consistency.*

**Response to Comment 10：**

Thank you for your careful observation. We have corrected all instances of “BCT” and “UCT” to the consistent terms “BCCT” and “UCCT” in Table 4. We have also carefully reviewed the entire manuscript to ensure that all terminology is now used consistently throughout. (Lines 311–314)

**Response to Comment 11：**

*L270: Please add bibliography.*

**Response to Comment 11：**

Thank you for your careful and detailed review. The sentence in question has been removed during the revision of the Discussion section. As this content is no longer present in the manuscript, no additional citation is needed.

**Reviewer's Comment 12:**

*L362: Authors should include in the study's limitations the absence of a control group that could have engaged solely in the volleyball training sessions.*

**Response to Comment 12：**

Thank you for this valuable comment. As suggested, we have included this point in the Limitations section (Lines 398–405). Specifically, we note that the absence of a passive control group receiving only standard volleyball training limits the ability to isolate the intervention effects from natural adaptations or external confounders. We also recommend that future studies incorporate non-intervention control groups to strengthen causal inference and enhance the interpretability of training effects.
