# Supplementary material for: Effects of unilateral and bilateral complex-contrast training on lower limb strength and jump performance in collegiate female volleyball players
Source: PLoS One. 2025 Jun 30;20(6):e0327237. doi: 10.1371/journal.pone.0327237 (PMC12208454; doi:10.1371/journal.pone.0327237)
Supplement: Attachment — Submitted filename: renamed_7ec62.docx [file pone.0327237.s005.docx]

Dear Editors and Reviewers:

Thank you for your letter and for the reviewers’ comments concerning our manuscript entitled **“Effects of Unilateral and Bilateral Complex-Contrast Training on Lower Limb Strength and Jump Performance in Collegiate Female Volleyball Players” (ID: PONE-D-25-07993)**. On behalf of my co-authors, we thank you for allowing us to revise our manuscript. These comments are all valuable and very helpful for revising and improving our paper, and they provide important guiding significance to our research. We have studied the comments carefully and have made corrections, which we hope meet your approval.

The revised portions are marked in red in the manuscript. In the response to the reviewer, the parentheses at the end correspond to the line numbers. The main corrections in the paper and the responses to the reviewer’s comments are as follows:

**Responds to the Reviewer #1:**

Thank you for your review and constructive feedback; no additional comments were provided.

**Responds to the Reviewer #2:**

**Response to General Comment:**

*Thank you for addressing most of my prior comments. I have some additional (and some repeated) comments that I believe need to be addressed further. Please note that these comments relevant to non-tracked changes version of manuscript.*

**Response to General Comment：**

Thank you for your continued evaluation and for identifying issues we had not previously noticed. Your insights have significantly improved the clarity and rigor of our manuscript. We have carefully addressed each additional and repeated comment below, with revisions noted by line number.

**Reviewer's Comment 1:**

*Line 21 – spss needs to be written in full. Although you could remove “in SPSS software” from the abstract as this information is in the methods.*

**Response to Comment 1：**

Thank you for your feedback. As suggested, we have removed “in SPSS software” from the abstract and ensured that “IBM SPSS Statistics” is written in full at first mention in the Methods section. (Line 21)

**Reviewer's Comment 2:**

*Line 24 – 1-RM needs to be written in full before using the abbreviation.*

**Response to Comment 2：**

Thank you for your comment. We have revised the manuscript so that “one-repetition maximum (1-RM)” is written in full at first mention. (Line 24)

**Reviewer's Comment 3:**

*Line 40 – suggest removing the word “level” from “vertical jump level”*

**Response to Comment 3：**

Thank you for your reminder. We have removed the word “level” so that the phrase now reads “vertical jump.” (Line 41)

**Reviewer's Comment 4:**

*you should note that many of the studies included in this review don’t use comparative interventions (i.e., some compare CCT to RT alone or plyometric activity alone), which makes it harder to determine its effectiveness. This is where you could also discuss Luders et al., 2024 and Schneiker et al., 2023 in a little more detail, as they are two studies that used a volume and intensity matched intervention like yours (albeit with a bilateral focus), rather than simply citing them above.*

**Response to Comment 4：**

Thank you for your continued feedback. We appreciate your insightful comments and have addressed each point below.We have expanded the Introduction to note that many prior CCT studies lack truly volume‐and‐intensity‐matched RT comparison groups, making it unclear whether observed benefits arise from potentiation or workload differences. We then describe Luders et al. (2024) and Schneiker et al. (2023) in greater detail, highlighting their matched designs and outcomes. (Lines 79–90)

**Reviewer's Comment 5:**

*Line 92 – this sentence does not really add anything meaningful and is really just a bunch of buzzwords. Suggest removing.*

**Response to Comment 5：**

We have removed the sentence as suggested. (Line 91)

**Reviewer's Comment 6:**

*Line 109 – in line with my earlier comments, I would suggest removing the hypothesis entirely as based upon your pre-registration it is clear this is not the hypothesis you had when designing the study. This looks like a clear example of HARKing.*

**Response to Comment 6：**

Thank you for your helpful suggestion. We have removed the hypothesis as suggested. (Lines 118)

**Reviewer's Comment 7:**

*Line 128 – write SPSS in full*

**Response to Comment 7：**

Thank you for your suggestion. We have updated the text to “using a random number-based randomization method in IBM SPSS Statistics 26.” (Line 135)

**Reviewer's Comment 8:**

*Line 131 – need to write all these abbreviations in full the first time they appear in the manuscript.*

**Response to Comment 8：**

Thank you for the suggestion. We have updated the sentence to: “Before and after the training intervention, participants underwent tests for one-repetition maximum (1-RM) squat, Countermovement Jump (CMJ), Squat Jump (SJ), Drop Jump (DJ), Eccentric Utilization Rate (EUR), and Standing Long Jump (SLJ).” (Lines 138–140)

**Reviewer's Comment 9:**

*Line 141 – It seems like you didn’t really think the sample size through here OR tried to find an effect size that firs your sample size. You used a study that added a power focused training program to normal skills training, and then compared it to a control group that only did normal skills training. This effect size is likely to be much larger than what you would expect to see when comparing two similar interventions (like your study). In future, suggest either being more thoughtful when you conduct your power calculation OR state that you used a convenience sample if that is what you did.*

**Response to Comment 9：**

Thank you for this valuable suggestion. We recognize that using an effect size of f = 0.5—sourced from a study comparing a power‐focused intervention to standard skills training—may overestimate the true effect when comparing two similarly structured CCT protocols. At the time of our power calculation, no directly matching study was available, so we used the closest proxy. We also recruited the maximum number of participants possible within our study period; the sample size limitation is noted in the Limitations section. We appreciate your guidance and will take greater care in selecting an appropriate effect size for future studies.

**Reviewer's Comment 10:**

*Line 158 – body mass doesn’t need to be capitalized.*

**Response to Comment 10：**

Thank you for catching that. We have corrected “Body Mass” to “body mass.” (Line 166)

**Reviewer's Comment 11:**

*Table 3 – suggest noting that the rest periods were in minutes in this table*

**Response to Comment 11：**

Thank you for this helpful suggestion. We have updated Table 3 to specify that all rest periods are measured in minutes. (Lines 199–200)

**Reviewer's Comment 12:**

*Line 211 – suggest changing “better” to “best”*

**Response to Comment 12：**

Thank you for this suggestion. We have replaced “better” with “best.” (Line 220)

**Reviewer's Comment 13:**

*Line 232 – suggest noting this was back squat and Bulgarian squat here*

**Response to Comment 13：**

Thank you for this suggestion. We have updated the text to:

“One-Repetition Maximum Squat: The testing methods for both the Back Squat and the Bulgarian Split Squat followed the guidelines from the NSCA Testing and Evaluation Manual (38).” (Lines 241–242)

**Reviewer's Comment 14:**

*Line 284 to 288 – I would suggest removing the commentary around larger effect sizes indicating better results here. The fact that there was no group x time interactions present between these variables would suggest that these differences are potentially due to chance (indeed, if you reported the 95% CI for these effect sizes we would expect to see some notable variation.*

**Response to Comment 14：**

Thank you for this suggestion. We have removed the commentary around larger effect sizes. (Line 294)

**Response to Comment 15：**

*Line 297 – can remove this sentence.*

**Response to Comment 15：**

Thank you for your suggestion. We have removed the sentence. (Line 300)

**Reviewer's Comment 16:**

*Line 302 – In figure 4 it looks like not all participants completed 1RM squat assessment. Can you please note why this is the case and report the numbers that did complete in text here.*

**Response to Comment 16：**

Thank you for pointing this out. All participants completed the 1-RM squat assessment at both pre- and post-testing. We have added a clarification to the Figure 4 caption to explain that overlapping points represent identical pre- and post-test values. (Line 311-312)

**Reviewer's Comment 17:**

*Line 302 – should also add 1RM Bulgarian split squat to the results assuming you tested it at the end of the intervention? Can also add it to table 5.*

**Response to Comment 17：**

Thank you for this question. The Bulgarian Split Squat 1-RM was assessed only at baseline for load prescription and, due to logistical constraints immediately following the intervention, was not repeated afterward; therefore, no post-intervention Bulgarian Split Squat data are available to include.

**Reviewer's Comment 18:**

*Line 318 to 321 – in line with one of my earlier comments, you can remove this as it may have been to chance.*

**Response to Comment 18：**

Thank you for this suggestion. We have removed the sentence as requested. (Line 324)

**Reviewer's Comment 19:**

*Line 383 – suggest removing this sentence, as you pre-registration didn’t have any hypotheses presented.*

**Response to Comment 19：**

Thank you for this suggestion. We have removed the sentence. (Lines 386–388)

**Responds to the Reviewer #3:**

Thank you for your constructive feedback; no additional comments were provided.
